# Supplementary material for: Deformation and dynamic response of abdominal aortic aneurysm sealing
Source: Sci Rep. 2017 Dec 18;7:17712. doi: 10.1038/s41598-017-17759-3 (PMC5735182; doi:10.1038/s41598-017-17759-3)
Supplement: Supplementary file 4 — Supplementary Information [file 41598_2017_17759_MOESM4_ESM.pdf]

Deformation and dynamic response of abdominal aortic aneurysm sealing

L.P. Argani[1*], F. Torella[2], R.K. Fisher[2], R.G. McWilliams[3], M.L. Wall[4], A.B. Movchan[1*]

SUPPLEMENTARY INFORMATION

Supplementary Information Guide

Supplementary Notes

Supplementary notes provide an additional explanation for Fig. 3, Table 1 (in the main text), and for the Supplementary Videos, presented in three files, which show vibration modes of a sealed abdominal aneurysm.

Supplementary Figures

Supplementary figures provide geometry informations, reference schemes and further results.

Supplementary Tables

Supplementary tables provide the physical parameters chosen for the modelling.

Supplementary Videos

Dynamic representation of Fig. 3 (in the main text), including two additional vibration modes and the first five vibration modes of the idealised geometry.

Supplementary Video Files

Supplementary Video 1. Sequence of vibration modes I to V of the idealised geometry in the case of endograft edges constrained at the bottom region of the sealed AAA.

Supplementary Video 2. Sequence of vibration modes I to V of Patient 1.

Supplementary Video 3. Sequence of vibration modes I to V of Patient 2.

Supplementary Notes

Vibration modes of the mathematical models for the idealised geometry (in the case of endograft edges constrained at the bottom) and for the real case studies (summarised in Fig. 3 in the main text) are illustrated in detail by means of Supplementary Videos showing the deformation of the aorta and the EVAS components occurring at the corresponding natural frequency; the corresponding frequency for each vibration mode presented in Supplementary Videos is reported in Table 1.

Because the eigenfrequency analysis does not provide the actual amplitude of the vibration modes but only their shape, the amplitudes of the motions shown in the Supplementary Videos are intentionally magnified to illustrate clearly the possibility of relative movements between the aorta and the endoprosthesis, as well as between EVAS components, induced by vibrations; similarly, to the same purpose of clarity, vibration modes are intentionally shown at the same arbitrary speed, although they refer to specific frequencies (the natural frequencies).

Supplementary Figures

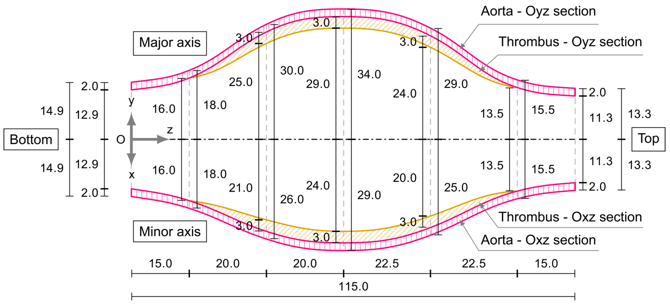

Supplementary Figure S1. Dimensions of the idealised model. All the measures reported are in millimetres.

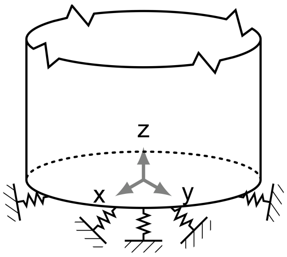

Supplementary Figure S2. Example of the spring-type boundary conditions. This scheme represents the end section of a generic component of the aorta-endoprosthesis sealing system subjected to continuity conditions.

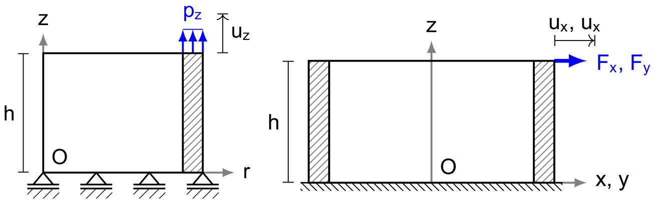

Supplementary Figure S3. Supplemental model for the calibration of the spring-type boundary conditions. Example of uniaxial tensile test (left) and shear test (right) on a tubular structure having the same cross section of the end section of an EVAS component subjected to continuity conditions.

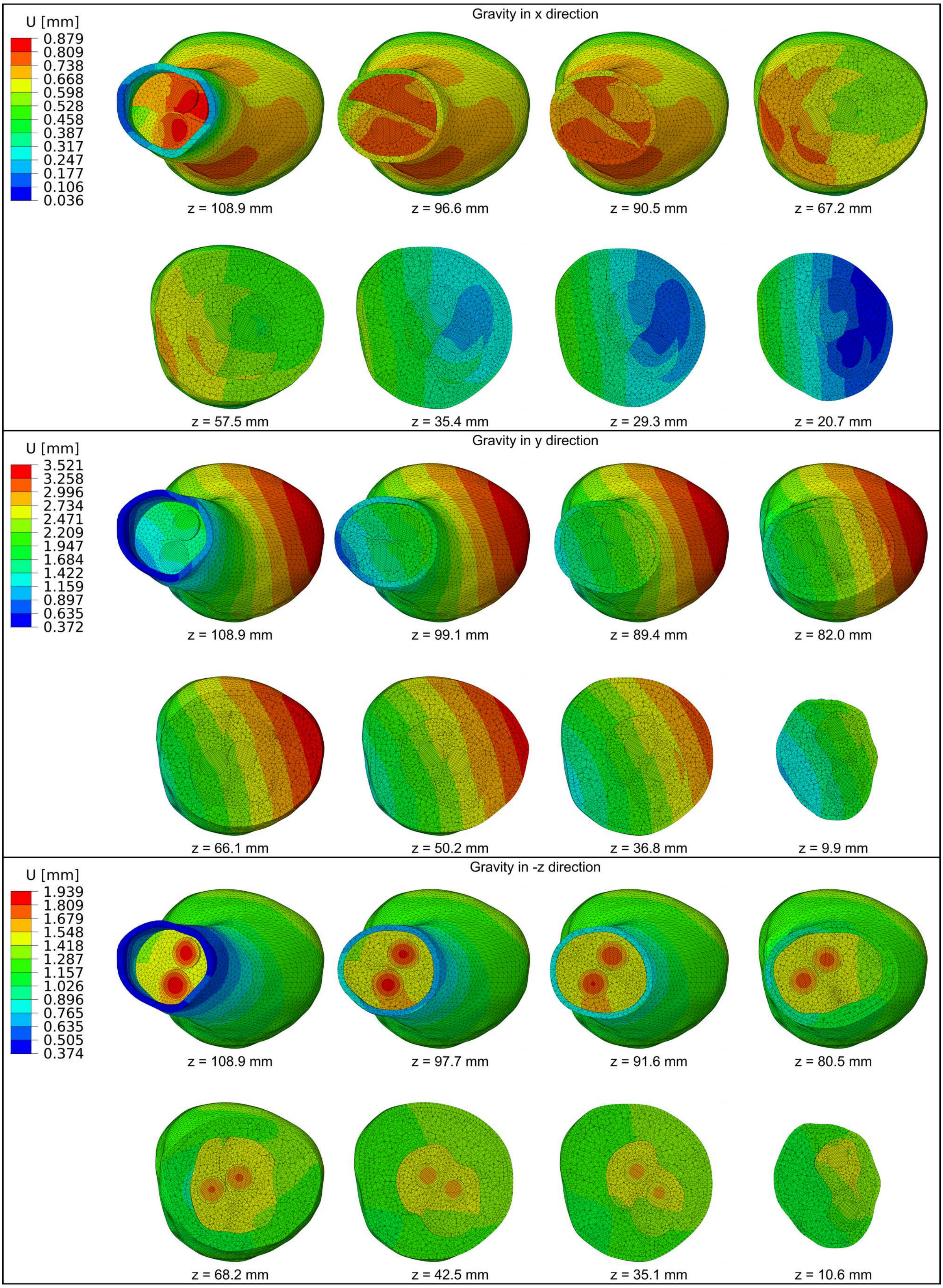

Supplementary Figure S4. Colour map of the displacement field magnitude U for Patient 1. Discontinuities (jumps) in the displacement field may occur only across the components interfaces (contact surfaces) and denote relative movements between the components of the system.

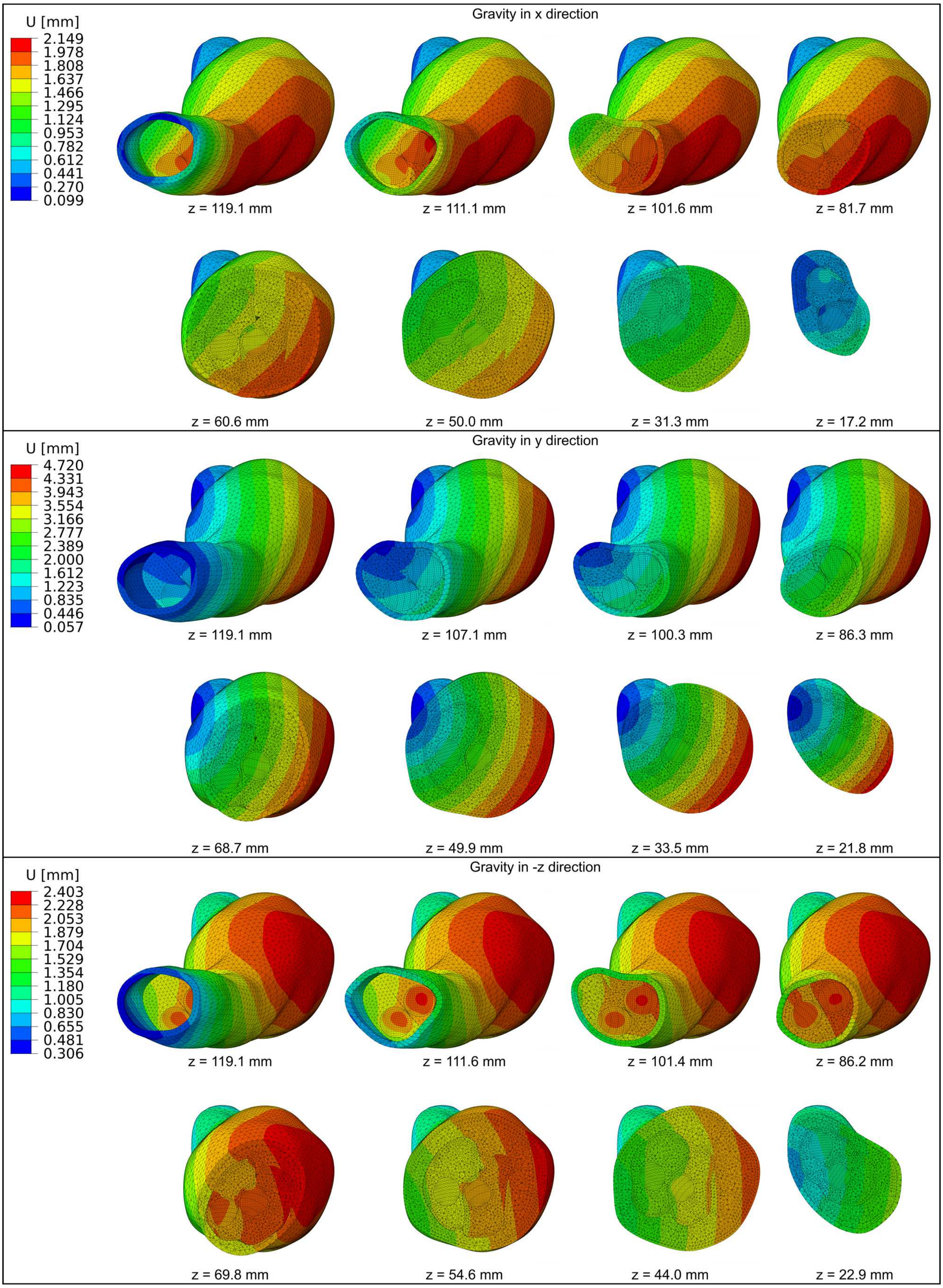

Supplementary Figure S5. Colour map of the displacement field magnitude U for Patient 2. Discontinuities (jumps) in the displacement field may occur only across the components interfaces (contact surfaces) and denote relative movements between the components of the system.

Supplementary Tables

Supplementary Table S1. Definition of the elastic properties of the materials employed in the mathematical model

Mechanical properties	Components of the AAA repair system			
	Aorta	Thrombus	Endobag	Graft
Young's modulus [MPa]	0.80	0.40	0.09	500
Poisson's ratio	0.49	0.45	0.49	0.46
Density [kg m^{-3}]	1200	910	2000	2200
